# Experimental Prototyping and Atomistic Modeling of Graphene Quantum Dot-Sensitized Solar Cells

**DOI:** 10.3390/ma19173566

**Published:** 2026-08-22

**Authors:** Łukasz Kaczmarek, Piotr Zawadzki, Kacper Szymański, Grzegorz Ulisiak, Alan Marciniak

**Affiliations:** Institute of Materials Science and Engineering, Lodz University of Technology, Stefanowskiego 1/15 St., 90-537 Lodz, Poland; lukasz.kaczmarek@p.lodz.pl (Ł.K.); piotr.zawadzki@p.lodz.pl (P.Z.); 223157@edu.p.lodz.pl (K.S.); 236177@edu.p.lodz.pl (G.U.)

**Keywords:** dye-sensitized solar cells, DSSC, graphene quantum dots, photovoltaics, nanotechnology

## Abstract

In the era of global energy transition, the development of third-generation photovoltaic technologies, such as dye-sensitized solar cells, has emerged as a paramount challenge in materials engineering. This study is dedicated to the synthesis and implementation of graphene quantum dots as eco-friendly sensitizers within DSSC architectures. The GQDs were synthesized via a microwave-assisted hydrothermal route using biodegradable organic precursors, providing a “green” alternative to conventional, toxic heavy-metal-based materials. The nanocrystalline structure and optoelectronic properties of the sensitizer were verified through UV-Vis and visual photoluminescence assessment. A focal point of this research was the optimization of the GQD concentration on the mesoporous surface of the titanium dioxide photoanode. Measurements were conducted utilizing a custom-designed experimental setup integrated with 3D-printed (FDM) components and an Arduino microcontroller, ensuring precise data acquisition under controlled illumination conditions (405–625 nm). The results indicated an optimal operational point at a fivefold dilution of the stock solution (0.4 g/dm^3^), which yielded the highest open-circuit voltage ($V_{\mathrm{oc}}$) of 545.4 mV under UV irradiation. The decline in photovoltaic performance observed at higher concentrations was attributed to excessive nanostructure agglomeration, which effectively blocked the mesopores of the semiconductor. Furthermore, the demonstrated high chemical capacitance of the system imparts electrochemical capacitor-like characteristics to the cell, enabling energy stabilization under fluctuating illumination. To elucidate the underlying sensitization mechanisms at the atomic level, computational simulations were conducted utilizing the MACE machine-learning potential and the GFN2-xTB semi-empirical method. The theoretical models revealed that the formation of stable covalent Ti–O–C bridges (chemisorption) is imperative for establishing strong interfacial electronic coupling. Solvation models and molecular dynamics (MD) at 300 K confirmed the thermodynamic and operational robustness of the hybrid system in an aqueous electrolyte. Ultimately, this combined experimental and theoretical work conclusively demonstrates that graphene quantum dots represent an efficient, highly stable, and non-toxic alternative to classic molecular dye sensitizers.

## 1. Introduction

Contemporary materials engineering is increasingly shifting toward nanoscale structures, among which graphene quantum dots occupy a prominent position. Graphene quantum dots (GQDs) are carbon nanostructures comprising isolated graphene fragments, typically sub-10 nm in size [1,2]. Owing to quantum confinement and pronounced edge effects, these materials exhibit unique physicochemical properties unobserved in their macroscopic counterparts. GQDs are classified as smart materials [3], as their optical and electronic responses can be precisely tuned by modifying their size [4], shape [5], or surface functionalization [6,7,8,9]. A key advantage of GQDs, distinguishing them from traditional quantum dots based on II-VI semiconductors, is their eco-friendly nature [10]. They are non-toxic [11] and exhibit excellent biocompatibility [12,13,14], effectively eliminating the environmental barriers associated with heavy metals such as cadmium or lead [15]. Building upon these crucial insights, our study leverages the specific surface chemistry of oxygen-rich GQDs to establish highly efficient charge-transfer pathways directly at the semiconductor interface. Furthermore, GQDs align perfectly with the principles of green chemistry, as they can be synthesized via cost-effective and highly efficient bottom-up routes using widely available organic precursors, including sugars, organic acids, or plant extracts [16,17,18,19]. This remarkable versatility has facilitated their widespread application in medical diagnostics [20,21], bioimaging [22,23], and as highly sensitive sensing platforms [24].

**Figure 1:**
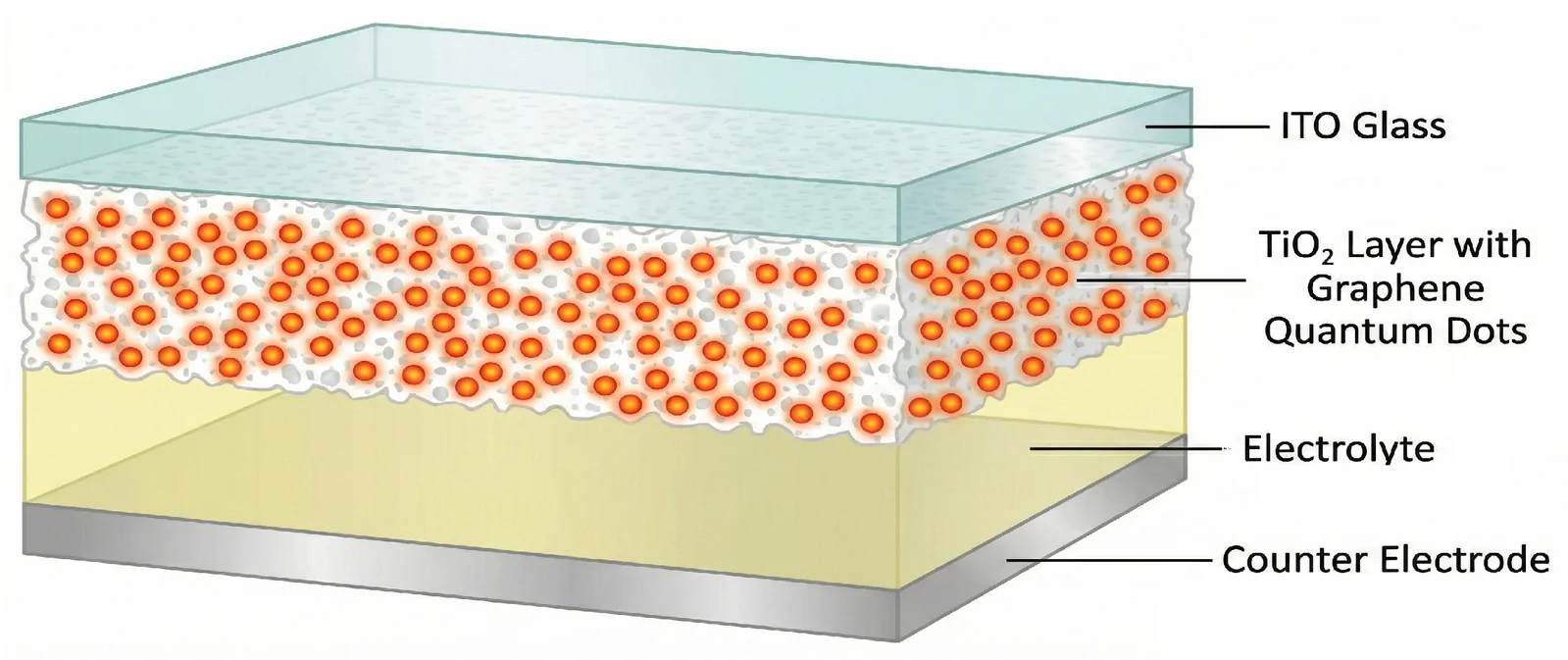
Schematic diagram of a GQD-sensitized Grätzel solar cell.This illustration was generated with the assistance of an AI tool (Google Gemini 3 Pro).

However, the potential of GQDs extends far beyond biomedicine, serving as a foundation for exploring novel solutions in sustainable energy [25]. Thanks to their capacity for broadband photon harvesting and ultrafast interfacial charge transfer, these materials are emerging as crucial components in third-generation photovoltaic technologies [26]. In the broader landscape of these emerging technologies, high-efficiency solar cells based on 1D engineered nanostructures (e.g., semiconducting nanowires or nanotubes) are currently challenging traditional architectures by providing directed charge transport pathways and enhanced light trapping [27,28]. Concurrently, another particularly promising field for the implementation of functional nanomaterials is dye-sensitized solar cells (DSSCs), also known as Grätzel cells [29]. Inspired by natural photosynthesis, the architecture of these devices relies on decoupling light absorption (performed by the sensitizer) from charge carrier transport within the mesoporous matrix of a wide-bandgap semiconductor, most commonly titanium dioxide (TiO_2_) [26]. A schematic representation of such a cell is depicted in Figure 1.

In DSSCs, GQDs can serve as highly stable, eco-friendly sensitizers, replacing expensive ruthenium complexes or easily degradable natural dyes. Recently, interfacial engineering strategies utilizing carbon-based sensitizers have emerged as highly promising avenues to overcome the limitations of traditional dyes. As demonstrated by recent advances in the field, targeted carbon-material-assisted interfacial regulation can dictate charge transport kinetics, suppress recombination, and enhance overall electronic and photocatalytic performance [30]. In the specific context of photovoltaic applications, carbon nanomaterials are currently at the forefront of this interfacial revolution. Recent theoretical and experimental studies demonstrate that integrating graphene-based derivatives directly into the TiO_2_ photoanode architecture alters quantum dynamics, providing ultrafast conductive pathways that overcome parasitic recombination processes [31]. Utilizing GQDs broadens the device’s absorption spectrum and improves the kinetics of electron injection into the TiO_2_ conduction band [32]. This study focuses on optimizing the implementation of GQDs, synthesized via a rapid microwave-assisted method, within the structure of Grätzel cells. The primary experimental objective is to demonstrate that the energy conversion efficiency in such systems is correlated with nanomaterial concentration, thereby minimizing agglomeration phenomena and maximizing the photoelectric activity of the active layer.

Furthermore, to elucidate the underlying interfacial mechanisms driving these photovoltaic metrics, the experimental efforts were coupled with computational modeling. Utilizing machine-learning potentials and semi-empirical quantum chemistry methods [33,34], this study investigates the thermodynamic and electronic differences between the physisorption and chemisorption of GQDs on the TiO_2_ surface. These atomistic simulations aim to uncover the nature of interfacial electronic coupling, evaluate the thermal and solvated stability of the resulting nanocomposite, and ultimately provide a theoretical framework justifying the experimentally observed DSSC performance. Ultimately, bridging the gap between theoretical calculations and practical device prototyping remains a significant challenge in the literature. By directly coupling advanced machine-learning simulations with an accessible, open-source experimental platform, this work moves beyond isolated computational or macroscopic studies. This integrated approach establishes a foundation for understanding interfacial charge dynamics, offering both a theoretical and practical framework for the future design of third-generation photovoltaics.

## 2. Materials and Methods

### 2.1. Materials for Cell Fabrication

**Figure 2:**
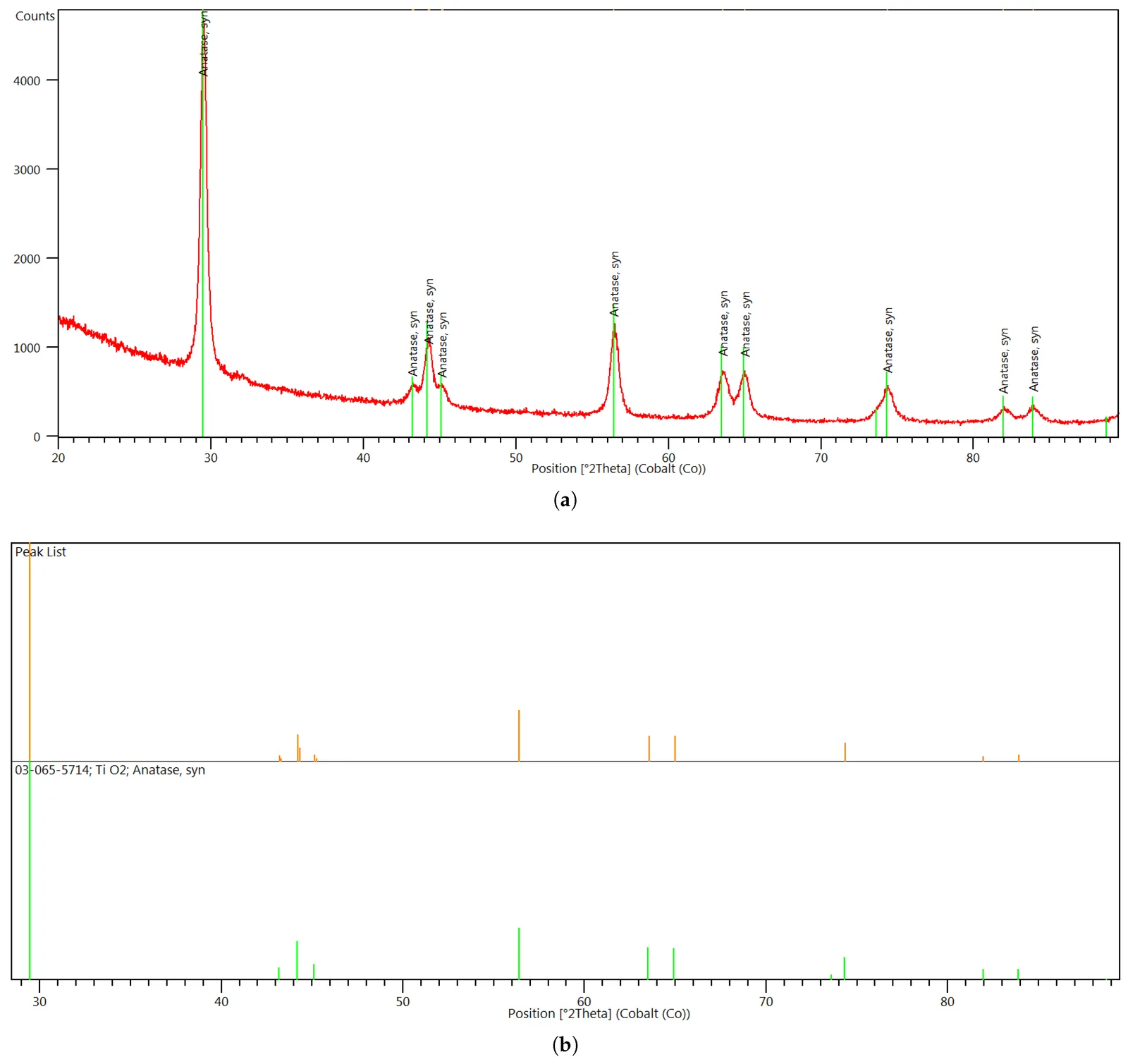
Structural characterization of the semiconductor layer: (**a**) The recorded XRD diffractogram of the TiO_2_ powder exhibiting reflections characteristic of the anatase phase; (**b**) list of reference peaks for anatase.

The structural foundation of the prototype solar cells consisted of 100×100 mm glass plates, coated on a single side with a transparent indium tin oxide (ITO) conductive layer (custom-supplied by Oknoplast, Ochmanów, Poland; proprietary physical specifications). Titanium dioxide powder was employed as the semiconductor material to fabricate the mesoporous photoanode, which was initially prepared as a colloidal suspension. Given the commercial origin of the raw material, its crystal structure and phase purity were verified via X-ray diffraction (XRD). The measurements were performed using a diffractometer equipped with a cobalt (Co) X-ray source. The diffractograms were recorded in the 2θ angular range from 20° to 90°. The acquired diffractograms (Figure 2) confirmed that the utilized semiconductor consists of pure anatase, with no measurable traces of rutile or brookite phases. The selection of this specific crystalline phase was dictated by its optimal charge transport properties and high adsorption capacity for graphene quantum dot-based sensitizers [35].

The counter electrode (cathode) was coated with a catalytic carbon layer in the form of graphite, deposited via a specialized aerosol consisting of a colloidal suspension of fine-grained graphite in a solvent mixture supplemented with a polymeric binder (Graphit 33, Kontakt Chemie, CRC Industries, Horsham, PA, USA). This formulation facilitated the deposition of a highly homogeneous coating featuring a substantial active area of 800 mm^2^ alongside high chemical stability. The role of the electrolyte, essential for dye regeneration and charge carrier transport, was fulfilled by an iodine/potassium iodide solution (Solutio Iodi Cum Glycerini, COEL, Kraków, Poland), which acted as an efficient $I^{-}/I_{3}^{-}$ redox couple.

### 2.2. Synthesis and Characterization of Graphene Quantum Dots

**Figure 3:**
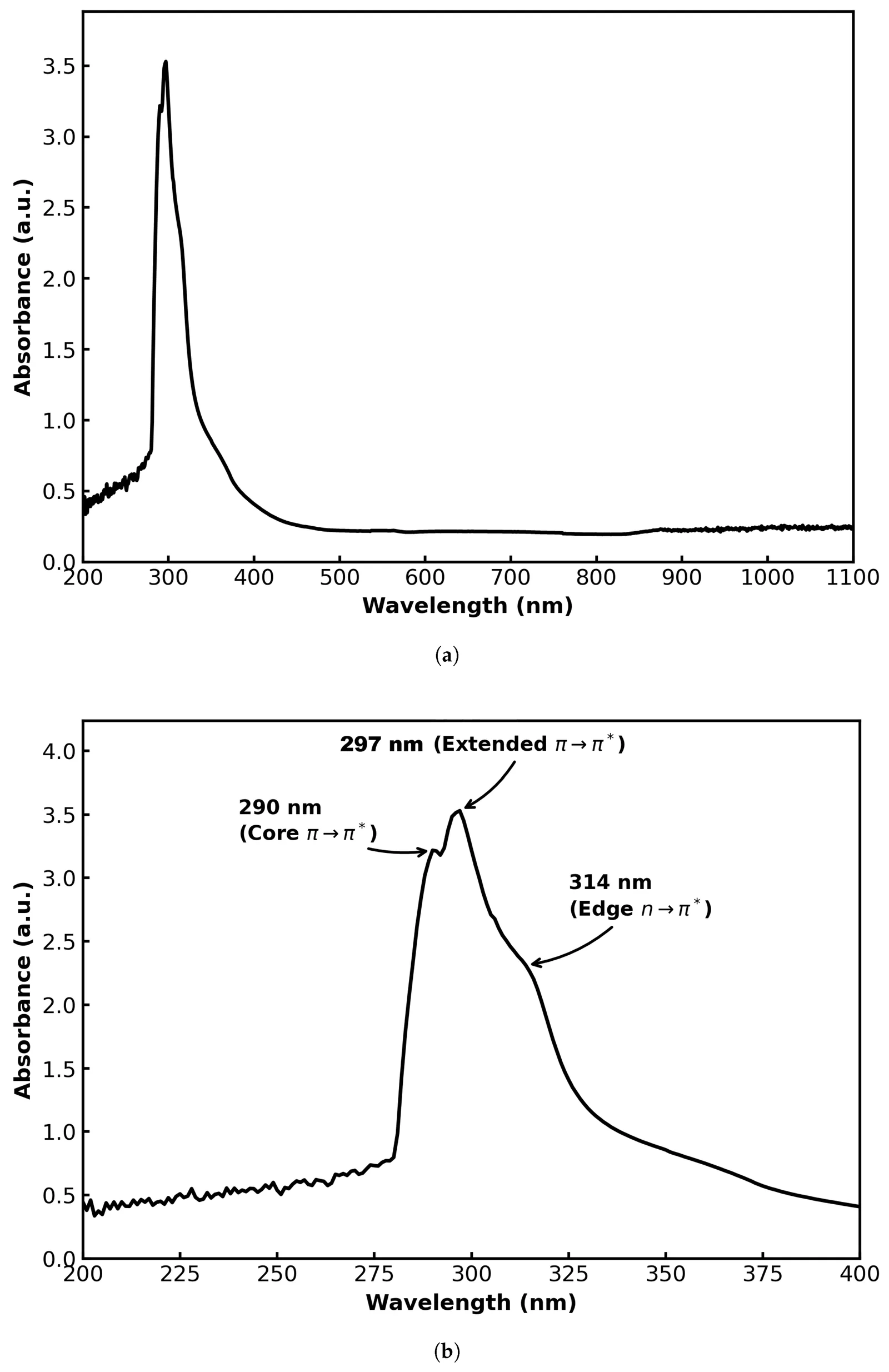
UV-Vis absorption spectroscopy of the synthesized graphene quantum dots: (**a**) full recorded spectrum; (**b**) magnified region between 200 and 400 nm.

**Figure 4:**
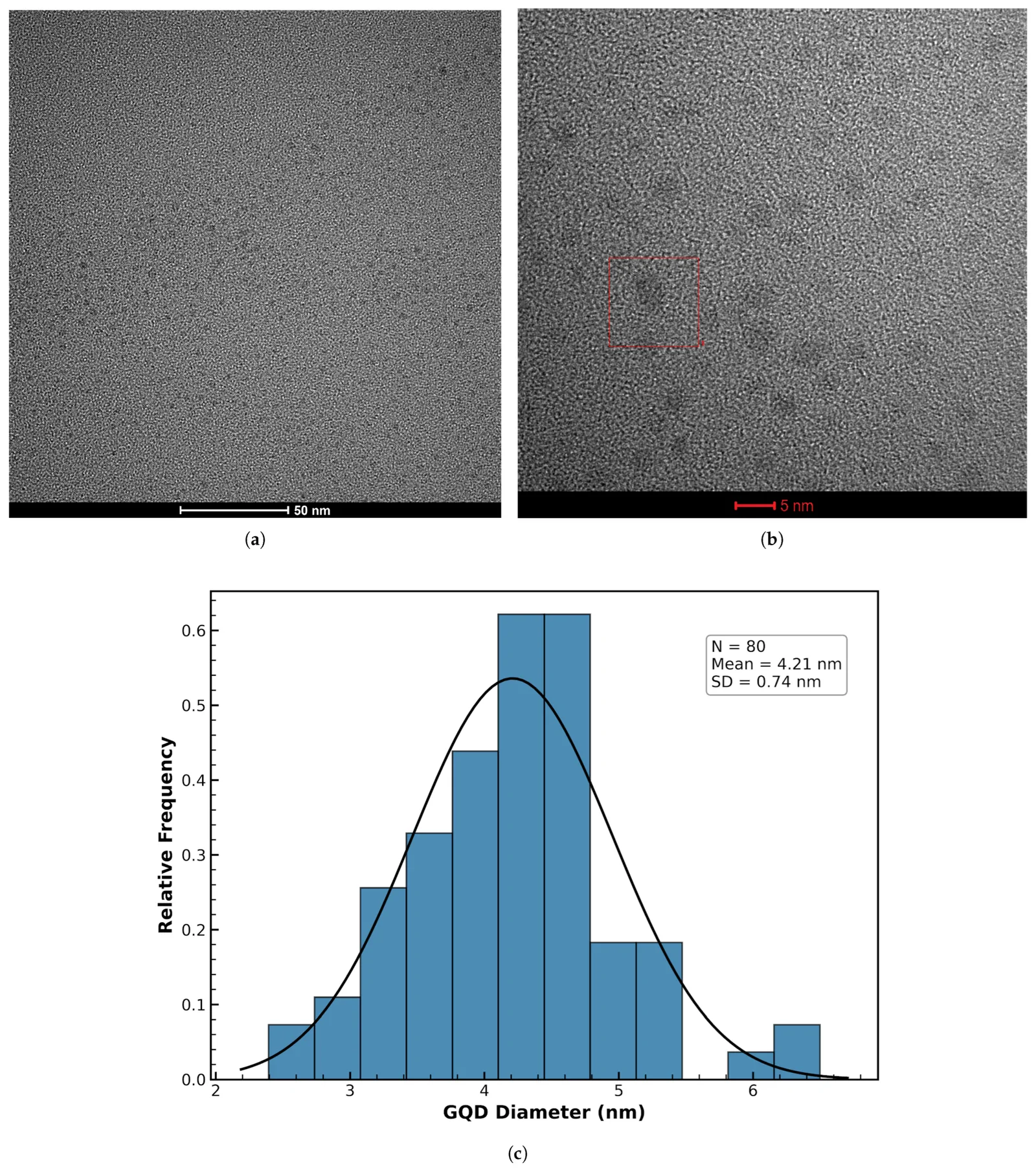
Morphology and size distribution of graphene quantum dots: (**a**,**b**) TEM images of the synthesized GQDs (the red box highlights an individual GQD); (**c**) statistical size distribution histogram derived from TEM analysis.

**Figure 5:**
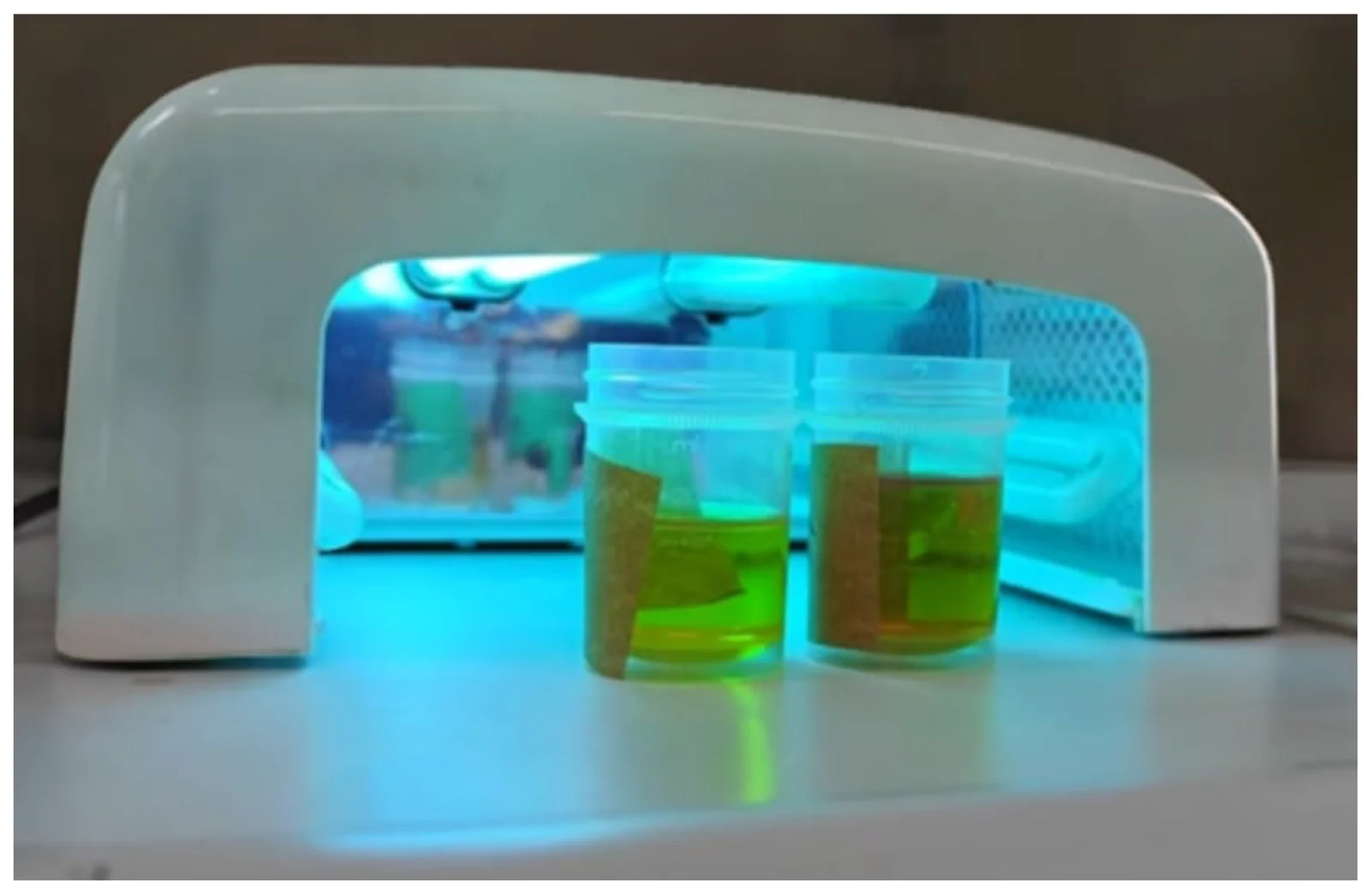
Photograph of the GQD colloidal solution exhibiting intense green photoluminescence under UV irradiation.

**Figure 6:**
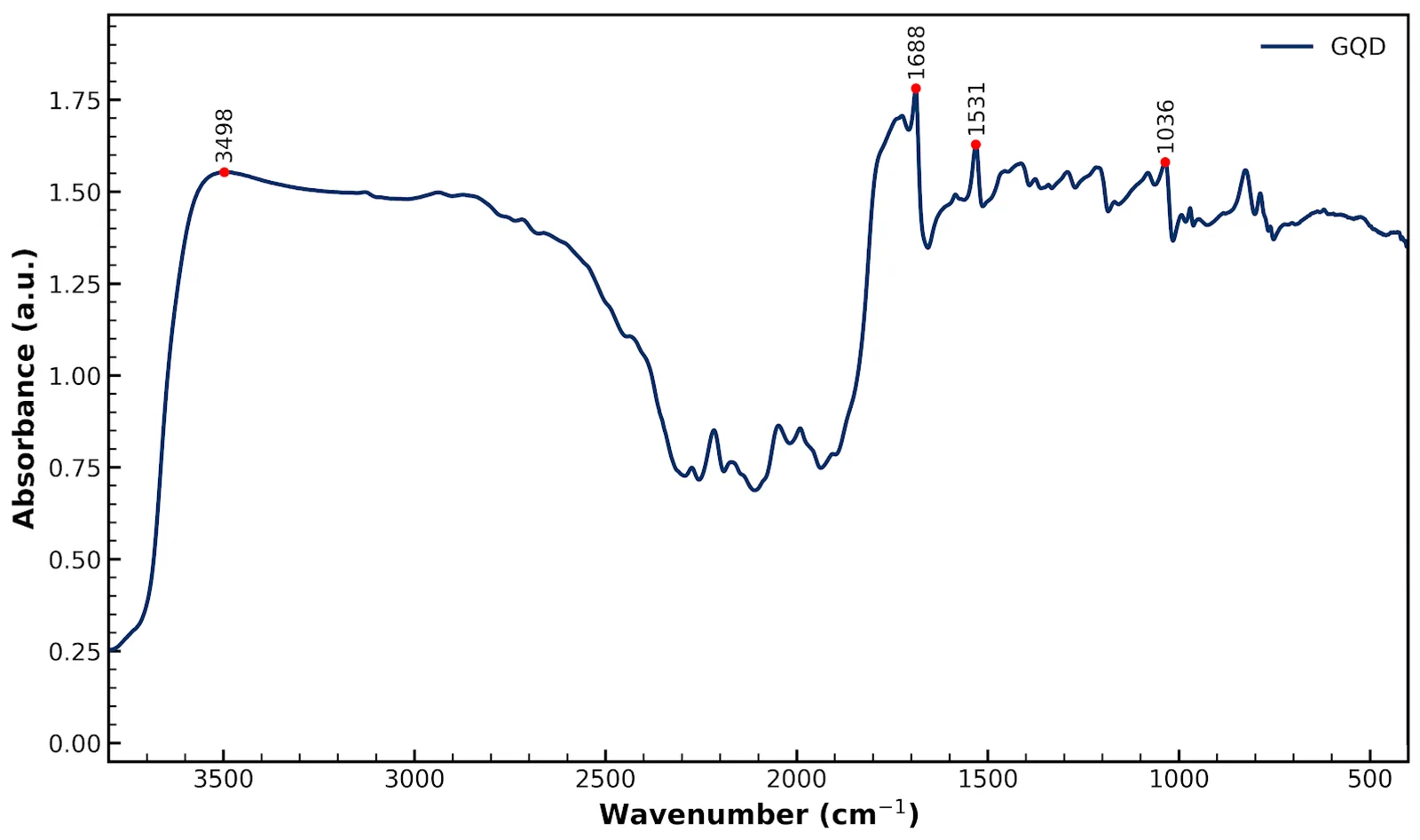
FTIR spectrum of the synthesized graphene quantum dots (GQDs).

Graphene quantum dots were synthesized via a microwave-assisted hydrothermal route utilizing biodegradable organic precursors. The specific chemical composition of the precursors, as well as the exact model of the reactor and detailed operational parameters, are currently the subject of a pending patent application and thus cannot be fully disclosed in this manuscript. This method facilitated the maintenance of a stable solution temperature, ensuring homogeneous nucleation and carbonization of the structures without the risk of precursor overheating. The mass concentration of the stock colloidal solution was determined via gravimetric analysis to be 2 g/dm^3^. Subsequently, a series of dilutions was prepared from this stock solution to optimize the performance of the fabricated solar cells. The identity of the synthesized nanostructures was confirmed via UV-Vis absorption spectroscopy (Figure 3a) utilizing a Shimadzu UV-2700i spectrometer (Shimadzu Corporation, Kyoto, Japan). To identify the specific electronic transitions underlying the broad absorption band, the ultraviolet region (200–400 nm) was isolated and analyzed in detail (Figure 3b). The experimental spectrum exhibits a tripartite absorption profile. The initial pronounced shoulder located at 290 nm is directly attributed to the $\pi \to \pi^{*}$ transition of the aromatic C=C bonds within the primary $sp^{2}$ hybridized graphitic core. The absolute absorption maximum, centered closely at 297 nm, represents an extended $\pi \to \pi^{*}$ transition. The emergence of this closely spaced doublet (290 nm and 297 nm) is characteristic of the quantum confinement effect or the electronic influence of the edge functionalization. Furthermore, the third feature appears as a broad inflection at 314 nm. This band originates from the $n\to \pi^{*}$ transition associated with oxygen-containing functional groups terminating the edges of the quantum dots. Identification of this transition profile corroborates the FTIR structural analysis, confirming both the heterogeneous carbonaceous core formation and the high degree of edge functionalization of the synthesized active nanomaterial [36]. Identification of these transitions corroborates the FTIR structural analysis, confirming both the carbonaceous core formation and the high degree of edge functionalization of the synthesized nanomaterial. The presence and morphology of the GQDs within the colloidal dispersion were further verified using transmission electron microscopy (TEM) imaging. The samples were prepared by drop-casting the GQD colloidal suspension onto a standard carbon-coated TEM grid, followed by solvent evaporation at room temperature. The microscopic observations were carried out using a transmission electron microscope (facility provided by Bionanopark, Łódź, Poland) operating at an accelerating voltage of 200 kV. Figure 4a,b illustrate the morphology of the dispersed graphene quantum dots. Statistical size distribution analysis derived from the manual measurement of the TEM micrographs (Figure 4c) reveals a narrow dimensional range, with the synthesized nanoparticles exhibiting an average diameter of 4.21 ± 0.74 nm (*N* = 80). This specific size regime confirms quantum confinement effects, which are responsible for the observed optoelectronic properties and the appropriate alignment of the frontier orbitals modeled in our chemical simulations. Qualitative optical validation was provided by the macroscopic observation of intense green photoluminescence under a UV lamp (Figure 5). It should be explicitly noted that rather than extracting quantitative spectrofluorimetric emission spectra, this macroscopic fluorescence was utilized as a rapid, reliable visual indicator. In the context of our experimental prototyping, this visual assessment sufficiently confirmed the successful carbonization and the formation of quantum-confined nanostructures prior to their immediate integration into the photoanode. To further elucidate the surface chemistry of the synthesized GQDs, Fourier-transform infrared (FTIR) spectroscopy was performed using a Thermo Scientific Nicolet iS50 FTIR spectrometer (Thermo Fisher Scientific, Waltham, MA, USA). The acquired spectrum (Figure 6) reveals characteristic absorption bands indicating the abundant presence of oxygen-rich functional groups. Specifically, the band centered at approximately 3498 cm^−1^ corresponds to the O–H stretching vibrations of hydroxyl groups and intercalated water molecules. Crucially, the sharp peak at 1688 cm^−1^ is assigned to the C=O stretching vibrations typical for carboxyl or carbonyl functionalities. Furthermore, the band at 1531 cm^−1^ relates to the skeletal C=C stretching of the aromatic graphene domains, while the peak at 1036 cm^−1^ is attributed to C–O stretching vibrations. The presence of these edge-functional groups experimentally confirms that the synthesized GQDs possess the necessary chemical anchoring sites required for effective coupling with the semiconductor surface.

### 2.3. DSSC Fabrication and Assembly Technology

The cell fabrication process was multi-stage and required precise surface preparation. Initially, the ITO-coated glass substrates underwent a degreasing procedure using acetone. The graphite counter-electrodes were prepared by sequentially spraying two layers of aerosol, with a 60 min interval between applications to allow for solvent evaporation, followed by 24 h stabilization at room temperature to form the required catalytic structure.

Simultaneously, the formation of the photoanodes was initiated. A titanium dioxide layer was deposited onto the conductive glass using the doctor-blade technique. The geometry and thickness of the layer were defined by masking with a thin tape (approximately 60 μm thick), which served as a guide. The active material was applied in the form of an aqueous suspension with a 1:3 weight ratio, ensuring a uniform layer thickness equal to the thickness of the masking tape, after which the layers were stabilized for 24 h.

**Figure 7:**
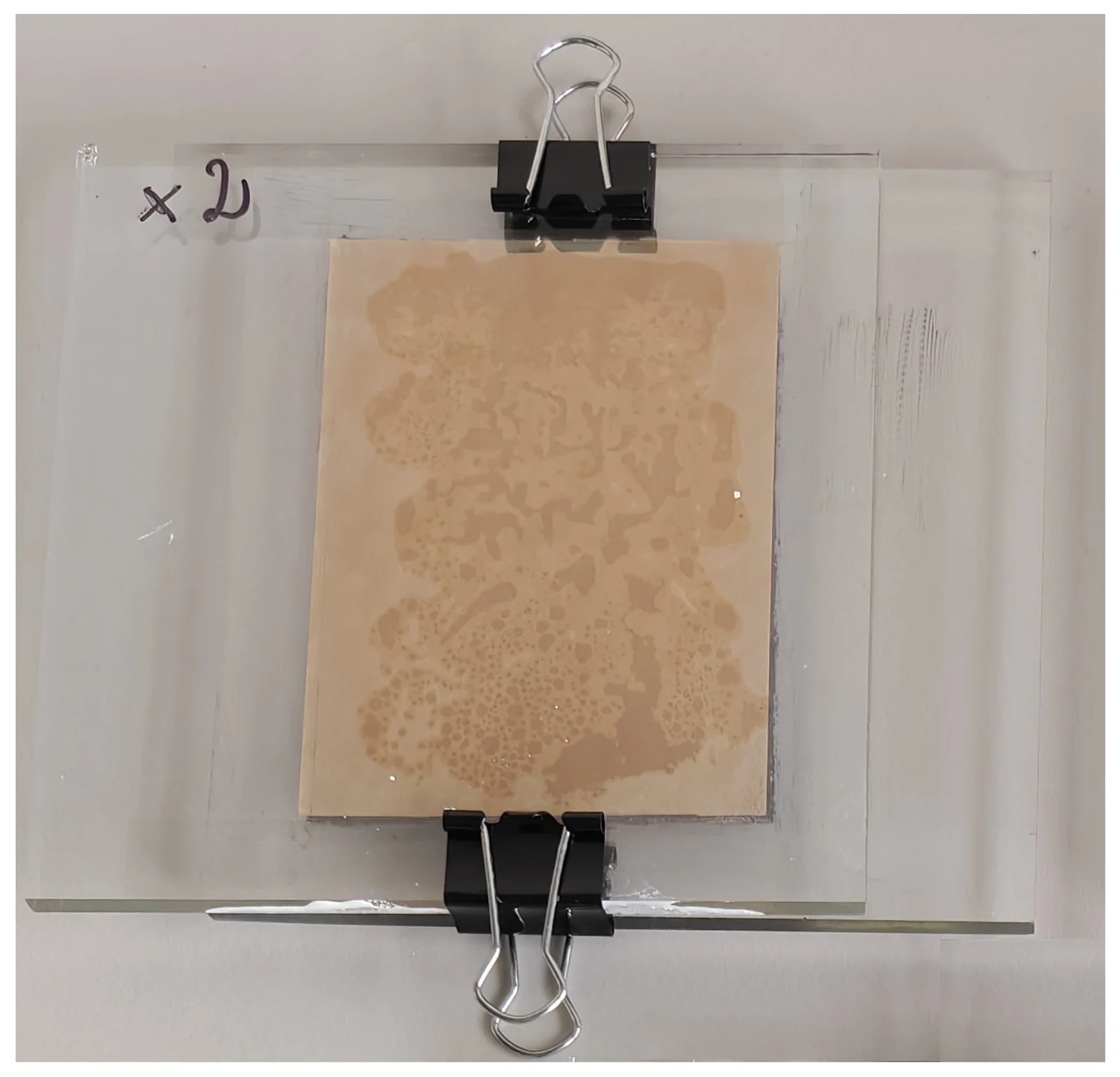
Assembled GQD-sensitized solar cell.

The sensitization stage involved soaking the mesoporous TiO_2_ structure with the prepared GQD solutions of varying concentrations; the samples were then left for 24 h to ensure the complete evaporation of the liquid phase. For comparative purposes, reference samples without a sensitizer were also prepared. A detailed description of the sample designations and GQD concentrations is provided in Table 1. The final device assembly was realized in a “sandwich” architecture. Both electrodes were assembled with their active layers facing inward, positioned so that the working areas overlapped completely. The entire assembly was mechanically stabilized using pressure clips. Subsequently, the electrolyte was introduced into the system, filling the pores of the semiconductor layer via capillary forces and ensuring direct electrochemical contact between the components. The fully assembled cell is illustrated in Figure 7.

### 2.4. Photoelectric Measurement Methodology

The performance characterization of the solar cells was conducted utilizing a custom-designed experimental setup functioning as an optical darkroom. The chamber, with dimensions of 300×300×150 mm, was equipped with a system of holders fabricated via 3D printing (FDM) technology using the Elegoo Neptune 4 Pro printer (Elegoo Inc., Shenzhen, China), ensuring the reproducible positioning of the cell at a precise distance of 100 mm from the light source. Interchangeable 0.1 W LED modules were utilized as emitters, generating light with dominant wavelengths of 405 nm (UV), 470 nm (blue), 520 nm (green), and 625 nm (red). The illumination sequence was managed by a control system based on an Arduino Uno microcontroller (Arduino, Ivrea, Italy). The acquisition of the open-circuit voltage was performed using a high-precision Fluke 115 digital multimeter (True RMS) (Fluke Corporation, Everett, WA, USA), enabling the reliable recording of the photoelectric response. The measurement protocol mandated the stabilization of the dark voltage ($V_{d}$) prior to the activation of the light source, followed by the averaging of a series of five independent readings for each specific wavelength.

### 2.5. Computational Methods

From a software engineering perspective, the entire simulation architecture was implemented utilizing the Atomic Simulation Environment (ASE) library [37]. ASE served as the overarching framework, managing system construction, atomic trajectories, and the optimization loops. To compute the forces and total energy of the complex systems, a hybrid computational approach was deployed. The primary quantum engine driving the simulations was the MACE machine-learning potential [33,38], configured in the “medium” variant with float32 precision to optimize computational runtime. The selection of MACE was dictated by its demonstrated capability to predict electrostatic energies, Pauli repulsion, and the breaking/formation of rigid covalent bonds with accuracy comparable to classical Density Functional Theory (DFT), yet at a fraction of the computational cost. However, since the MACE model, trained predominantly on databases utilizing the PBE functional, exhibits recognized deficiencies in capturing long-range van der Waals forces, the architecture was augmented with the *simple-dftd3* library [39]. This module implements Grimme’s third-generation empirical dispersion correction (D3), which is essential for accurately capturing long-range van der Waals forces that standard computational potentials often underestimate. Furthermore, it is coupled with Becke–Johnson (BJ) damping, a mathematical function that prevents non-physical infinite attraction at very short interatomic distances, ensuring a smooth and realistic potential energy surface. These two methods were dynamically coupled in real time utilizing the SumCalculator tool within the ASE package (version 3.28.0). To ensure the D3 algorithm could correctly evaluate the stress tensor and volume for the isolated carbon quantum dot, the structure was embedded within a virtual, orthorhombic vacuum zone with bounding edge lengths of 40 Å.

In both adsorption scenarios, a bi-layer anatase slab served as the geometric foundation of the system. The slab was cleaved along the (101) crystallographic plane, which represents the most thermodynamically stable and frequently exposed facet of this mineral. The fundamental unit cell was replicated to construct a substantial 9×6×1 molecule supercell, generating an extensive surface comprising around 1000 titanium and oxygen atoms. This area provided sufficient spatial clearance to prevent the graphene dot from interacting with its own periodic images under the applied Periodic Boundary Conditions (PBC).

The carbon quantum dot in the control variant (physisorption) was modeled as an idealized, defect-free graphene fragment featuring “armchair” edges, containing approximately 70 carbon atoms, with all edge valencies fully saturated by hydrogen. Conversely, for the chemisorption simulation, this exact dot underwent targeted functionalization. Two adjacent hydrogen atoms were computationally removed from a designated edge and substituted with hydroxyl (-OH) and carboxyl (-COOH) functional groups, preserving their natural, unconstrained spatial geometry [40].

The computational protocol in both experiments relied on a thermodynamic framework. Initially, the algorithm executed independent geometry optimizations for the isolated oxide substrate and the isolated carbon dot. The structures were allowed to relax to their local energetic minima using the LBFGS (Limited-memory Broyden–Fletcher–Goldfarb–Shanno) algorithm. LBFGS is a highly efficient mathematical optimization procedure that iteratively adjusts atomic positions to find the most stable, lowest-energy geometry without requiring the computationally expensive calculation of the full Hessian matrix. This structural relaxation was governed by a stringent force convergence criterion set at 0.01 eV/Å. This means the optimization procedure stopped only when the residual forces acting on any individual atom fell below this near-zero threshold, guaranteeing that the final structures represent a stable thermodynamic equilibrium. These fully relaxed, unstrained structures subsequently established the energetic baseline for calculating the final binding energies.

To thoroughly explore the configurational space, three distinct initial spatial arrangements were constructed for each dot type, yielding a total of six modeled systems. In the first setup, the GQD was positioned parallel (face-on) to the anatase substrate at a standard van der Waals distance. This configuration served as a baseline to assess non-covalent physisorption interactions. In the second setup, the parallel orientation was maintained, but the interfacial distance was artificially reduced to 2.0 Å. This forced-proximity model was designed to probe the steric repulsion and the potential for spontaneous bonding across the entire carbon basal plane. The third setup employed a tilted geometry, with the carbon framework inclined at a 45° angle relative to the surface plane. The quantum dot was positioned such that its lowest-lying atom (an edge carbon in the pristine model, and an oxygen atom of the functional group in the functionalized model) was placed at a reactive distance of 2.0 Å directly above a surface titanium atom. This angled initialization was chosen to minimize the steric hindrance of the basal plane while facilitating direct orbital overlap at the interface. Following the spatial initialization, all six configurations were subjected to full, unconstrained geometric optimization to determine the final anchoring geometries and calculate the corresponding adsorption energies.

The starting point for the quantum calculations consisted of the spatially optimized composite geometries, previously acquired via MACE with the D3 dispersion correction. Due to the prohibitive computational costs associated with the quantum-mechanical description of the entire periodic system, the well-established cluster model approach was employed [41]. Rather than analyzing an infinite crystal lattice, the optimized structure was reduced to a localized cluster, selectively isolating the carbon quantum dot along with its immediate chemical environment (titanium and oxygen atoms within the direct anchoring zone).

To map the activation mechanisms of the graphene quantum dot as a sensitizer, an auxiliary simulation was performed to evaluate the impact of deprotonation on the nanocrystal’s electronic structure. These specific quantum calculations were executed utilizing the semi-empirical Extended Tight-Binding (xTB) framework [42], intentionally modeling the quantum dot with a net negative charge to simulate the detachment of a proton (H^+^) from its edge functional groups. Given the architecture of the investigated photovoltaic cell, which utilizes an aqueous solution of iodine and potassium iodide as the electrolyte, supplementary simulations were conducted to capture the environmental influence of the solvent on the sensitizer’s energy levels. For this purpose, the analytical linearized Poisson–Boltzmann (ALPB) implicit solvation model for water was integrated into the xTB calculations [43].

Furthermore, standard geometry optimizations performed at absolute zero (0 K) inherently neglect lattice thermal fluctuations, which are an intrinsic component of environments where DSSCs are implemented. To verify the operational stability of the synthesized composite, molecular dynamics simulations were executed at room temperature (300 K). These atomic trajectories were propagated using MACE coupled with a Langevin thermostat. The Langevin thermostat is a well-established algorithm in molecular dynamics used to maintain a constant macroscopic temperature by simulating the effect of a background heat bath. It achieves this by applying a precise combination of frictional drag and random thermal forces to the atoms, effectively mimicking the realistic kinetic energy fluctuations and lattice vibrations present in an operational solar cell. To ensure an accurate representation of the interfacial interactions between the graphene plane and the metal oxide, these thermal calculations were simultaneously augmented with the aforementioned van der Waals dispersion corrections. This procedure aimed to evaluate the thermodynamic resilience of the sensitizer against thermal desorption under continuous room-temperature agitation.

A generative artificial intelligence tool (Google Gemini 3 Pro) was utilized during the preparation of this manuscript to assist with language editing, stylistic improvements, and the generation of schematic illustrations (Figure 1).

## 3. Results and Discussion

### 3.1. Photoelectric Performance of the Fabricated Cells

**Figure 8:**
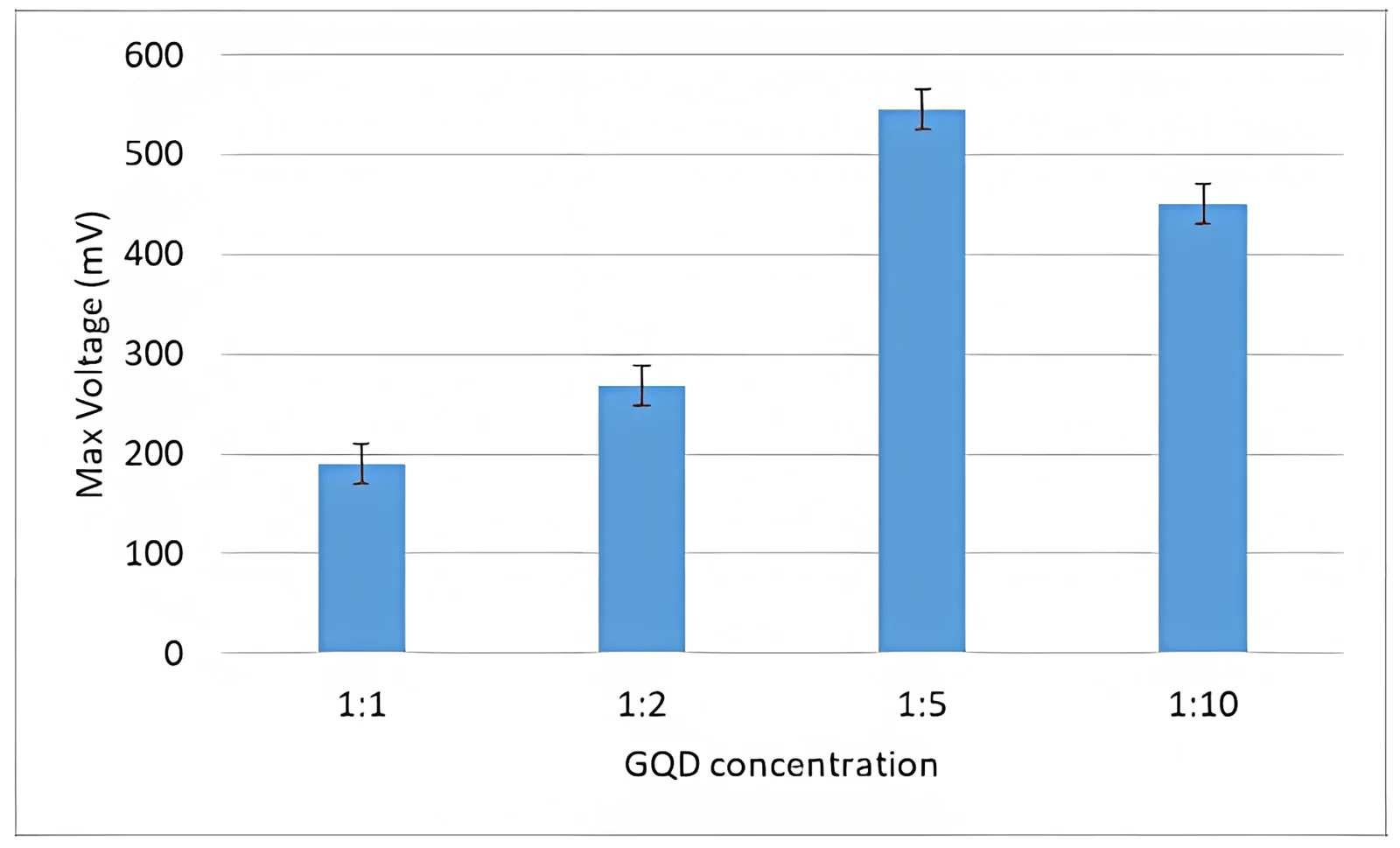
Maximum voltage in relation to the concentration of GQDs.

The measurements confirmed the generation of a macroscopic open-circuit voltage in all cells sensitized with graphene quantum dots, even in the absence of external illumination. The evaluated cells are denoted by their sensitizer dilution ratios (as detailed in Table 1): 1:1 (undiluted stock solution, 2.0 g/dm^3^), 1:2 (1.0 g/dm^3^), 1:5 (0.4 g/dm^3^), and 1:10 (0.2 g/dm^3^). This voltage, referred to as the dark voltage ($V_{d}$), was dependent on the concentration of the nanomaterial adsorbed onto the photoanode. The highest dark voltage was recorded for samples with a fivefold dilution of the stock solution (501 mV) (Figure 8), while the lowest values were observed for cells sensitized with the undiluted stock solution (102 mV). In the case of the control cell without a sensitizer, no voltage generation was detected under darkroom conditions.

**Figure 9:**
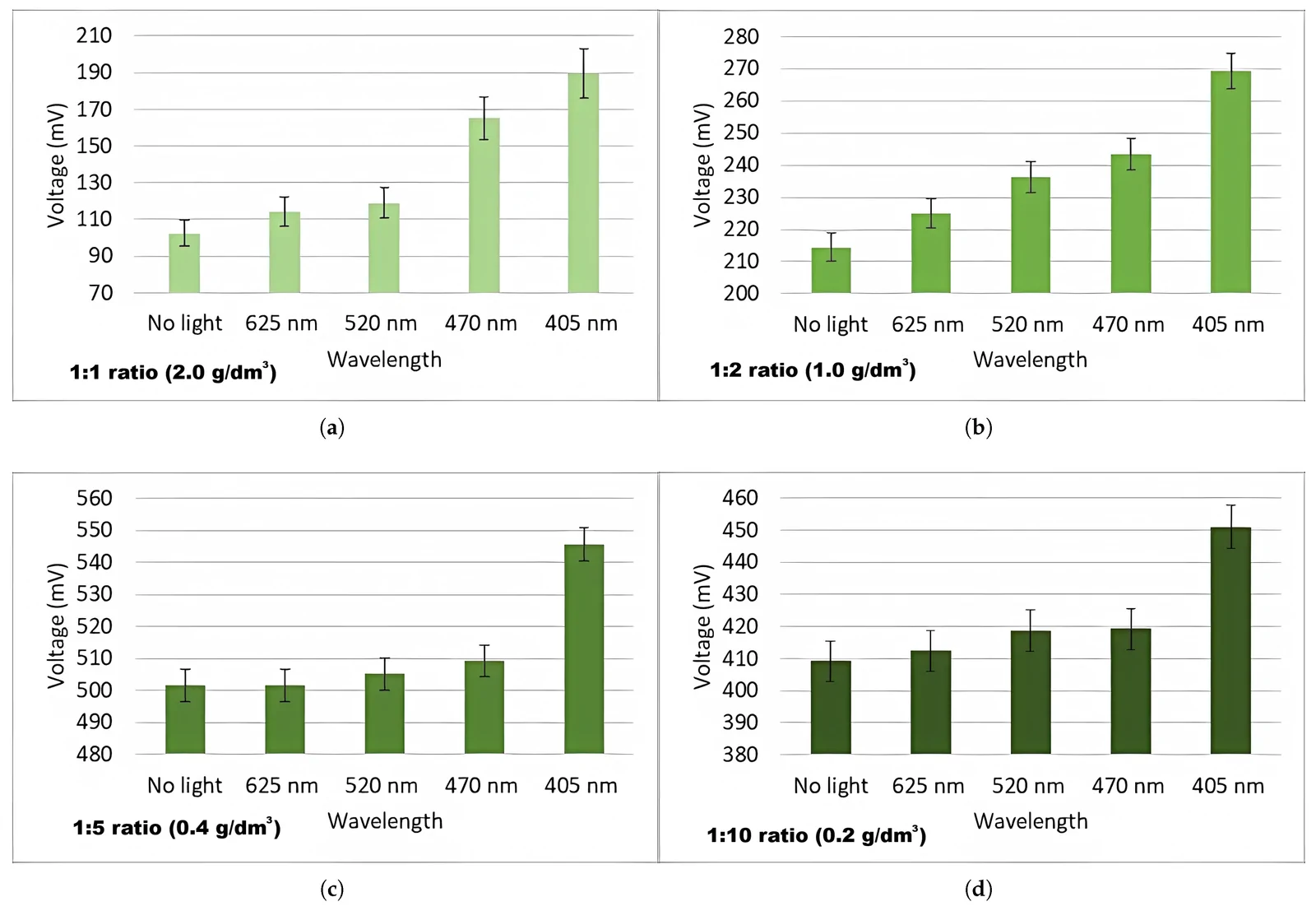
Open-circuit voltage ($V_{\mathrm{oc}}$) in relation to the illumination wavelength for varying GQD concentrations: (**a**) 1:1 ratio (2.0 g/dm^3^); (**b**) 1:2 ratio (1.0 g/dm^3^); (**c**) 1:5 ratio (0.4 g/dm^3^); and (**d**) 1:10 ratio (0.2 g/dm^3^).

**Figure 10:**
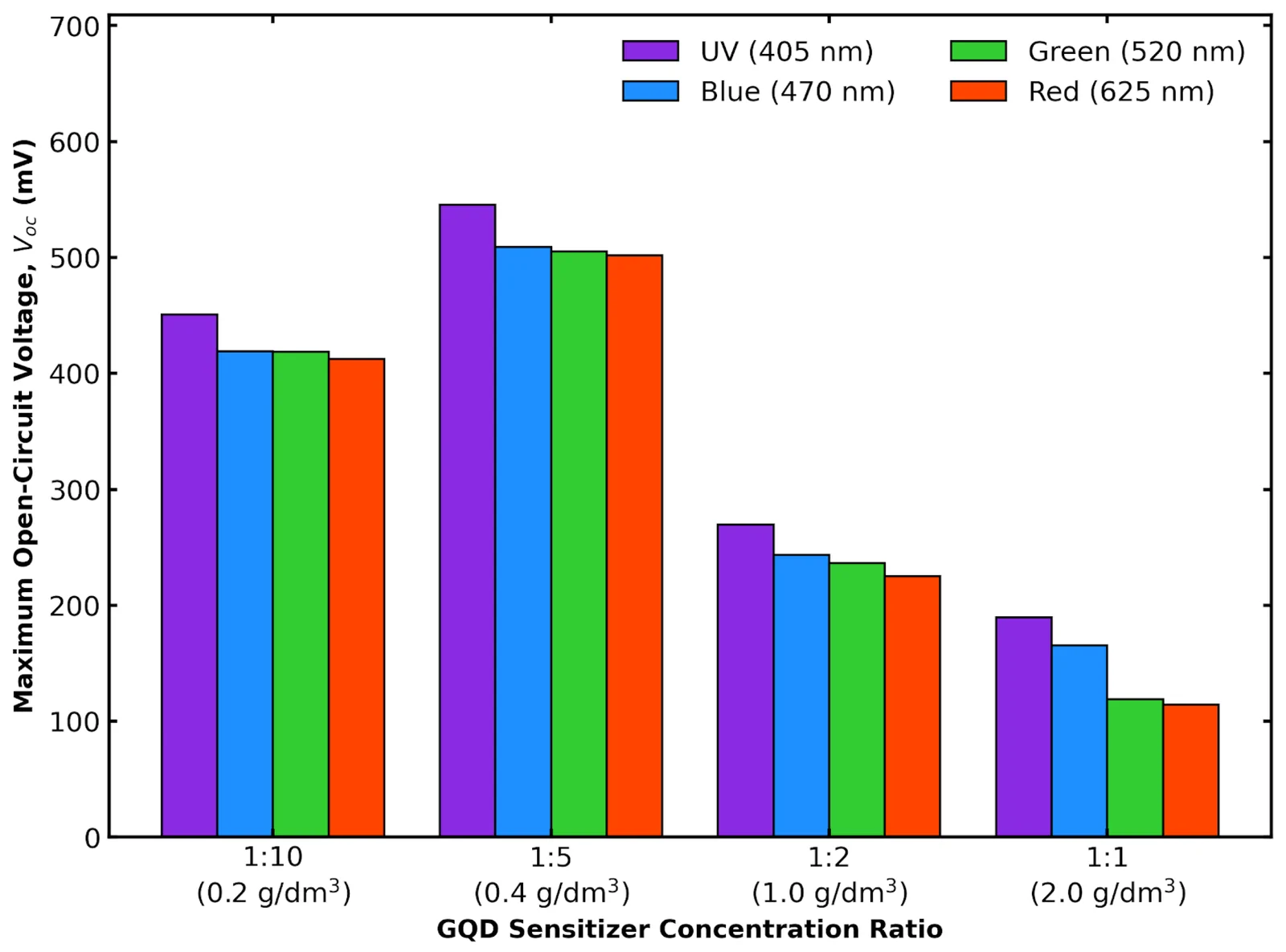
Summary bar chart of the maximum open-circuit voltage generated under specific illumination wavelengths as a function of the GQD sensitizer concentration ratio.

Upon activation of the light sources, an increase in the open-circuit voltage relative to the baseline values was observed in all cells containing the GQD sensitizer, whereas the reference cell (pure TiO_2_) remained photoelectrically inactive. The kinetic evolution of these responses under varying illumination is presented in Figure 9. To provide a comprehensive overview of the overall performance, a summary bar chart of the maximum $V_{\mathrm{oc}}$ versus GQD dilution ratio for each specific illumination wavelength is depicted in Figure 10. As shown, a consistent, wavelength-independent trend emerges: the photovoltage peaks at the 1:5 ratio across all tested light sources, achieving a maximum of 545.4 mV under UV irradiation. This response to ultraviolet excitation aligns with the primary optical absorption band of the GQDs identified in the UV-Vis spectra (Figure 3b). Conversely, the macroscopic photoelectric response decreases for longer wavelengths (blue, green, and red), mirroring the diminishing absorption cross-section of the carbon nanostructures in the visible regime. Crucially, the decline in $V_{\mathrm{oc}}$ at higher sensitizer concentrations across all individual wavelengths confirms that excessive GQD loading induces detrimental quenching and pore-blocking effects, irrespective of the incident excitation energy.

It should be noted that the present experimental configuration, utilizing a custom-designed Arduino-based acquisition system and monochromatic LED arrays, was purposefully engineered to monitor the kinetics of the open-circuit voltage rather than to extract standardized full current–voltage (J-V) curves. Consequently, the extraction of parameters such as short-circuit current density ($J_{sc}$), fill factor (FF), and power conversion efficiency (PCE) under standard AM 1.5G illumination falls outside the scope of this initial proof-of-concept study. Here, the macroscopic $V_{\mathrm{oc}}$ generation serves as a qualitative experimental validation for the advanced MACE/xTB quantum-chemical models. Comprehensive electrochemical characterizations are scheduled for our forthcoming investigations utilizing fully scaled standard solar simulators.

### 3.2. Capacitance of the Dye-Sensitized Solar Cells

The analysis of the photoelectric characteristics of the fabricated structures confirmed the unique properties of graphene quantum dots as sensitizers in DSSC systems. One of the most significant observations was the recording of a high and stable dark voltage ($V_{d}$), reaching approximately 500 mV for the 1:5 sample. This phenomenon confirms the high chemical capacitance of the mesoporous titanium dioxide layer, resulting from charge accumulation in trap states located within the semiconductor’s energy gap. In contrast to classical junction-based solar cells, the nanostructured TiO_2_/GQD system exhibits characteristics similar to an electrochemical capacitor, where accumulated electrons are slowly released, enabling the maintenance of a measurable potential even upon termination of illumination [44,45,46].

### 3.3. Impact of GQD Concentration on Voltage Generation

Based on the investigated dilutions, the optimal nanomaterial concentration was determined to be a fivefold dilution of the stock solution. This configuration yielded the highest open-circuit voltage of 545 mV under UV radiation. The observed decline in photovoltaic performance at higher GQD concentrations (1:1 and 1:2) indicates the occurrence of excessive nanostructure agglomeration on the photoanode surface. An over-density of GQDs promotes the formation of multilayer aggregates, which not only physically block the mesoporous TiO_2_ network, but also introduce deleterious optical and electronic effects. Specifically, excessive GQD loading leads to parasitic light shielding and concentration-driven fluorescence quenching. Furthermore, multilayer stacking increases the physical distance between the outermost sensitizer molecules and the semiconductor surface. This spatial separation impedes electron injection and simultaneously promotes enhanced charge recombination at the interface, leading to an increased internal resistance and a drop in the generated voltage [29,47]. A blank test performed on the unsensitized TiO_2_ layer unequivocally confirmed that the recorded photoelectric activity is a direct result of the carbon nanomaterial implementation, paving the way for further optimization of these structures in terms of energy conversion efficiency. However, it is crucial to emphasize that while strong chemisorption is a fundamental prerequisite for effective sensitization, there is no simple linear correlation between the absolute magnitude of the adsorption energy and the ultimate macroscopic performance of the DSSC. Although interfacial coupling guarantees necessary electron injection, excessive interfacial binding or an over-accumulation of strongly anchored sensitizers can introduce critical secondary bottlenecks. Specifically, an overly dense chemisorbed layer may sterically hinder electrolyte diffusion within the mesoporous TiO_2_ network and severely impede the requisite regeneration of the photo-oxidized sensitizer by the redox mediator. Furthermore, excessively strong electronic coupling might not only accelerate forward injection but also facilitate parasitic back-electron transfer from the semiconductor conduction band. Therefore, the successful macroscopic operation of the device relies on a delicate kinetic balance—the edge-functionalized GQDs provide the requisite anchoring stability and orbital overlap for primary injection, while careful experimental optimization of their concentration is necessary to prevent the detrimental transport and recombination effects associated with excessive interfacial saturation [29].

### 3.4. Structural Validation and Adsorption Thermodynamics

**Figure 11:**
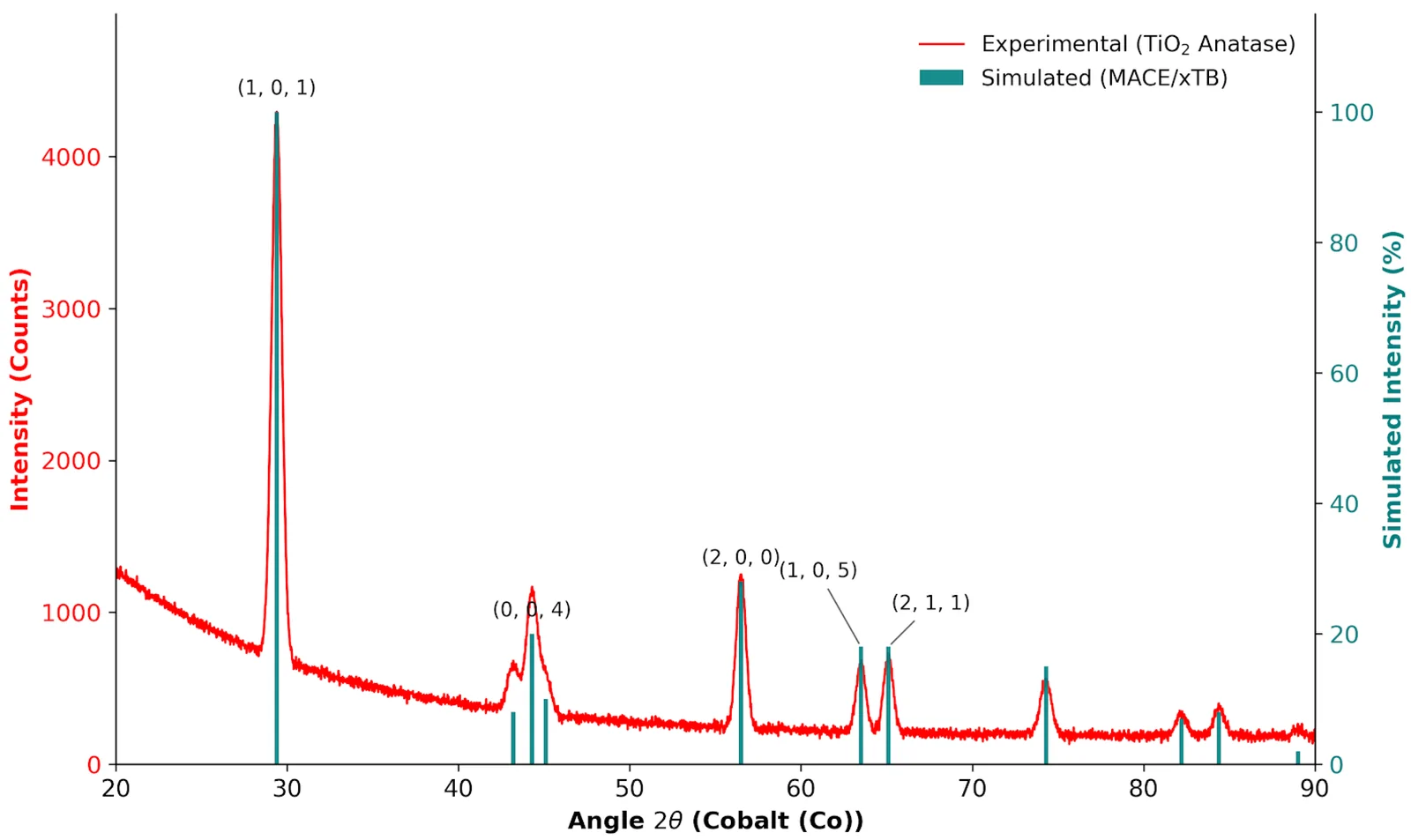
Comparative analysis of the X-ray diffraction patterns. Experimentally recorded diffractogram of the TiO_2_ powder overlaid with the theoretical XRD pattern simulated for the optimized anatase supercell.

To ensure the physicochemical reliability of the simulations, it was crucial to verify that the numerically generated oxide substrate replicates the actual crystal lattice of the material. To this end, a theoretical XRD pattern was simulated for the optimized TiO_2_ structure and overlaid with the experimentally recorded diffractogram of the utilized semiconductor material (Figure 11). The XRD simulation was performed using the *pymatgen* software package (version 2026.3.23) [48], with the radiation source configured to match the wavelength of a cobalt anode. The algorithm calculated the analytical peak profiles based on Bragg’s law (λ=2dsinθ), which defines the conditions for constructive wave interference across the lattice planes. Concurrently, atomic scattering factors were employed to determine the relative intensities of the individual reflections. These calculations relied on the standard Cromer–Mann scattering factor tables for neutral titanium (Z=22) and oxygen (Z=8) atoms. Ultimately, based on these diffraction conditions, the 2θ angles and normalized peak intensities were derived.

As shown in Figure 11, the simulated diffraction peaks align precisely with the experimental 2θ positions, confirming the structural accuracy of the anatase model. The inherent discrepancies (the peak broadening and background noise present in the experimental curve) are expected results of the nanoscale crystallite size and standard instrumental scattering, which are naturally not captured by the idealized periodic simulation. Overall, this geometric agreement successfully validates the computational model for subsequent adsorption studies.

**Figure 12:**
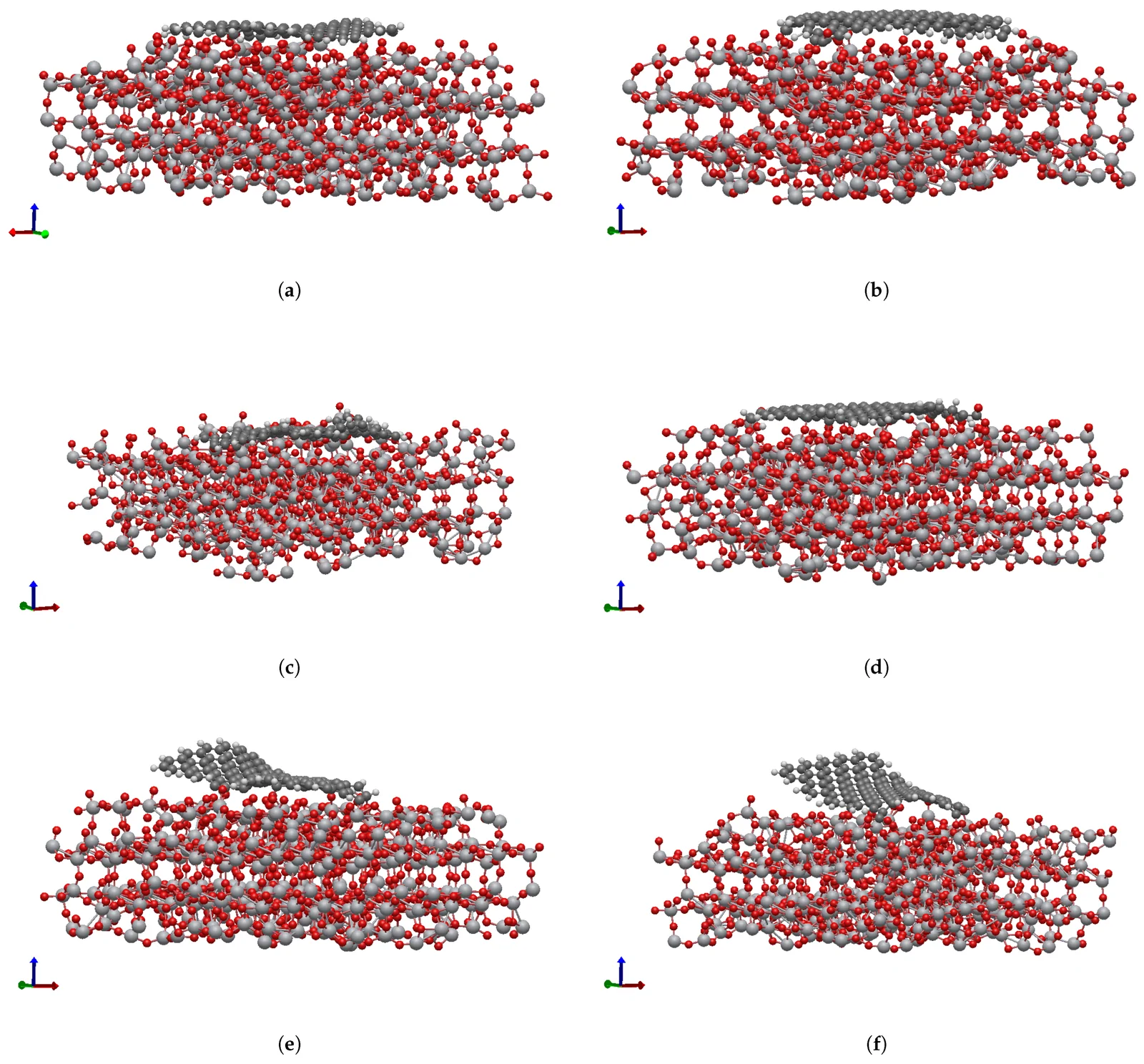
Optimized geometries of the investigated graphene quantum dot configurations on the anatase TiO_2_ (101) surface. The left column represents pristine GQDs, while the right column represents functionalized GQDs. (**a**) Pristine GQD, parallel orientation at van der Waals distance. (**b**) Functionalized GQD, parallel orientation at van der Waals distance. (**c**) Pristine GQD, parallel orientation at a forced 2.0 Å interfacial distance. (**d**) Functionalized GQD, parallel orientation at a forced 2.0 Å interfacial distance. (**e**) Pristine GQD, 45° tilted orientation at a 2.0 Å anchoring distance. (**f**) Functionalized GQD, 45° tilted orientation at a 2.0 Å anchoring distance. (Dark grey spheres represent carbon atoms, red spheres represent oxygen atoms, white spheres represent hydrogen atoms, and light grey spheres represent titanium atoms).

Following the geometric optimization of the six modeled configurations, the calculated adsorption energies provided a quantitative basis for analyzing the interfacial interactions. The results revealed thermodynamic regimes governed by both the presence of edge functionalization and the initial spatial orientation. In the parallel configurations initialized at a standard van der Waals distance, the systems exhibited weak dispersion interactions characteristic of physisorption. The pristine and functionalized GQDs yielded adsorption energies of −2.1 eV and −1.7 eV, respectively. These values align with theoretical reports indicating that pristine carbon frameworks interact with TiO_2_ primarily through weak van der Waals forces [49]. When the interfacial distance in the parallel orientation was artificially reduced to 2.0 Å, thermodynamic divergence was observed. The pristine GQD yielded a positive energy of +4.6 eV, indicating severe steric repulsion between the extended carbon basal plane and the TiO_2_ surface lattice, which results in an energetically unfavorable state. In contrast, the functionalized GQD at the same forced-proximity distance demonstrated substantial thermodynamic stabilization, reaching an adsorption energy of −20.5 eV. This observation is consistent with recent studies demonstrating that oxygen-containing edge groups form highly stable covalent bonds with transition metal oxides, overcoming the steric repulsion typical for pristine graphene [50]. The 45° tilted configurations further clarified the relationship between steric strain and optimal bond geometry. For the pristine GQD, inclining the carbon framework effectively relieved face-on repulsion, allowing the system to relax into a local minimum of −4.6 eV. However, the system achieved its absolute global minimum when the functionalized GQD was introduced at a 45° angle, yielding an adsorption energy of −20.7 eV. The slight energy difference (ΔE≈0.2 eV) between the tilted and parallel chemisorbed states indicates that the angled geometry minimizes residual basal strain while optimizing the bond angles of the newly formed ester bridges [51]. Overall, the energy disparity of over 16.0 eV between the optimally oriented functionalized and pristine dots serves as quantitative proof of successful chemical anchoring [52,53]. All relaxed models are shown in Figure 12. While the experimental FTIR analysis (Figure 6) confirms the presence of reactive -COOH and -OH groups on the GQD edges, directly probing the buried Ti–O–C interfacial bonds in assembled mesoporous devices via in operando spectroscopic methods remains inherently challenging. Consequently, computational modeling has become the state-of-the-art methodology for elucidating atomistic interfacial charge-transfer mechanisms in DSSCs [41,54]. By utilizing the experimentally validated edge-functionalized structure, our MACE-driven theoretical models bridge this resolution gap. Consistent with recent theoretical studies demonstrating the critical role of covalent grafting between carbon nanomaterials and transition metal oxides [49,50], our computational results suggest that the formation of stable Ti–O–C bridges is the primary mechanistic driver behind the macroscopic photovoltaic enhancement observed in our functional DSSC prototypes. Geometric and thermodynamic differences between the physisorbed and chemisorbed models explain the mechanism by which the functionalized carbon quantum dot operates as an effective sensitizer. The formation of the stable ester bridges, confirmed by adsorption energy in the tilted configuration, indicates the occurrence of interfacial electronic coupling between the carbon nanocrystal and the semiconductor substrate. These resulting surface bonds act as direct molecular bridges that facilitate rapid charge transfer from the dot to the metal oxide. This structural phenomenon directly accounts for the photoluminescence quenching observed in the literature for such hybrid systems [55]. Upon photon absorption and subsequent electron excitation to the quantum dot’s higher energy levels, the charge carrier does not undergo internal non-radiative or radiative recombination. Instead, exploiting the aforementioned chemical bridges and direct orbital overlap, it is instantaneously injected into the conduction band of titanium dioxide [56]. This finding aligns with recent literature emphasizing that targeted surface engineering and interfacial coupling of graphene quantum dots can effectively regulate carrier migration kinetics and enhance overall catalytic and electronic performance [57]. Specifically, the designed oxygen-rich functionalities on our GQDs act as dedicated charge-transfer channels, minimizing interfacial recombination and directing the electron flow toward the metal oxide. Conversely, a physisorption-based model relies exclusively on weak dispersion interactions. Lacking direct physical charge-flow pathways, this spatial separation would force the excited electrons into an inefficient tunneling process across the interface. In a functional device, such delayed transfer significantly increases the probability of thermal recombination. Consequently, the computational structural analysis proves that the geometric relaxation of the edge-functionalized dot, leading to stable covalent chemisorption, is crucial to prevent energy losses and ensure efficient electron injection in wide-bandgap semiconductor architectures [58,59]. It is important to address the substantial magnitude of the calculated adsorption energy for the optimal chemisorbed state. Such a highly exothermic value suggests the concurrent formation of multiple covalent or coordinate bonds between the densely packed oxygen functional groups on the GQD edge and the adjacent titanium atoms on the anatase surface, acting synergistically to anchor the dot. Furthermore, this absolute value must be interpreted within the methodological context of the employed machine-learning interatomic potential. While the MACE framework provides unprecedented computational efficiency for exploring large configurational spaces and accurately predicting global geometric relaxations, its absolute thermochemical outputs may deviate quantitatively from high-level DFT calculations. Machine-learning potentials can occasionally overestimate absolute binding energies at highly reactive complex interfaces. Such overestimations can be directly attributed to inherent biases in the density functional approximations used to generate the foundational training data. Furthermore, calculation methodology constraints, such as the finite model size of the simulated supercell and the dense packing of highly reactive functional groups undergoing simultaneous multi-bond formation, can artificially inflate the absolute exothermic response. Therefore, while the large relative energy disparity of over 16.0 eV between the pristine and functionalized models serves as semi-quantitative proof of the chemisorption mechanism and interfacial stabilization, the absolute binding energy values should be treated as illustrative. To obtain a strictly quantitative thermochemical profile of this interfacial hybridization, subsequent computational verification employing rigorous first-principles DFT calculations is highly recommended for future studies [33,60].

### 3.5. Effect of Deprotonation on the Sensitization Potential

Under realistic operational conditions, the anchoring of dyes onto metal oxide surfaces frequently involves the detachment of a proton (H^+^) from the edge functional groups, yielding a structure with a net negative charge. To evaluate this effect for the investigated system, additional simulations were performed on the isolated functionalized GQD in both its neutral and deprotonated states. The quantum chemical analysis demonstrated that the generation of this anionic state drastically modifies the positioning of the frontier orbitals [61]. Compared to the neutral quantum dot, where the vacuum-calculated HOMO and LUMO levels were situated at approximately −9.2 eV and −9.1 eV, respectively, the deprotonated system exhibited a sharp upward shift. The new HOMO was established at −6.7 eV, accompanied by a corresponding elevation of the LUMO to −6.6 eV. This substantial relative upshift in the energy levels (approximately 2.5 eV for both the HOMO and the LUMO) is driven by strong intramolecular Coulombic repulsion induced by the excess electron. From the perspective of device engineering, this coordinated shift is crucial for the optimization of photovoltaic cell performance. Elevating the energies of the frontier orbitals (particularly the LUMO) enhances the electron-donor potential of the carbon nanocrystal. Consequently, a much more favorable energy band alignment is established relative to the TiO_2_ conduction band. The augmented potential difference between the excited state of the quantum dot and the acceptor states of the substrate maximizes the thermodynamic driving force responsible for charge carrier injection, translating into greater energy conversion efficiency within the photocatalytic composite [47,62]. It should be noted that the notably narrow absolute HOMO–LUMO gap (ΔE≈0.1 eV) obtained in these specific configurations is a known computational artifact inherent to the GFN-xTB formalism. Base tight-binding models are spin-restricted and do not account for the spin-polarization of localized electron states at the graphene edges. As established in the literature, neglecting this spin-unrestricted radical character artificially collapses the energy gap, yielding a pseudo-metallic state rather than a true semiconductor [63,64]. Nevertheless, because this systematic underestimation affects both the neutral and deprotonated structures uniformly, the relative energy shift in the frontier orbitals upon deprotonation (∼2.5 eV) remains a reliable metric.

### 3.6. Influence of the Electrolyte Environment on Energetic Stabilization

The implicit solvation calculations revealed an impact of the electrolyte environment on the energetic stabilization of the deprotonated, negatively charged graphene quantum dot. Under reference vacuum conditions, excess charge induces strong intramolecular repulsion, situating the HOMO level at approximately −6.7 eV. However, the introduction of a continuous dielectric model of water (ε=78.4) triggered a profound downward shift in the frontier orbital energies by approximately 2.0 eV, stabilizing the HOMO at −8.7 eV. This phenomenon stems directly from the electrostatic shielding of the dot’s anionic charge by the surrounding solvent dipoles, which dissipates the excess energy. The downward energetic shift thermodynamically facilitates the reduction in the oxidized sensitizer by accepting electrons from a redox mediator (e.g., iodide ions, I^−^) present in the electrolyte. Consequently, adequate solvation ensures the uninterrupted continuity of the photocatalytic cycle while minimizing interfacial voltage losses between the dye and the electrolyte [65,66].

### 3.7. Thermal and Operational Stability of the Composite

**Figure 13:**
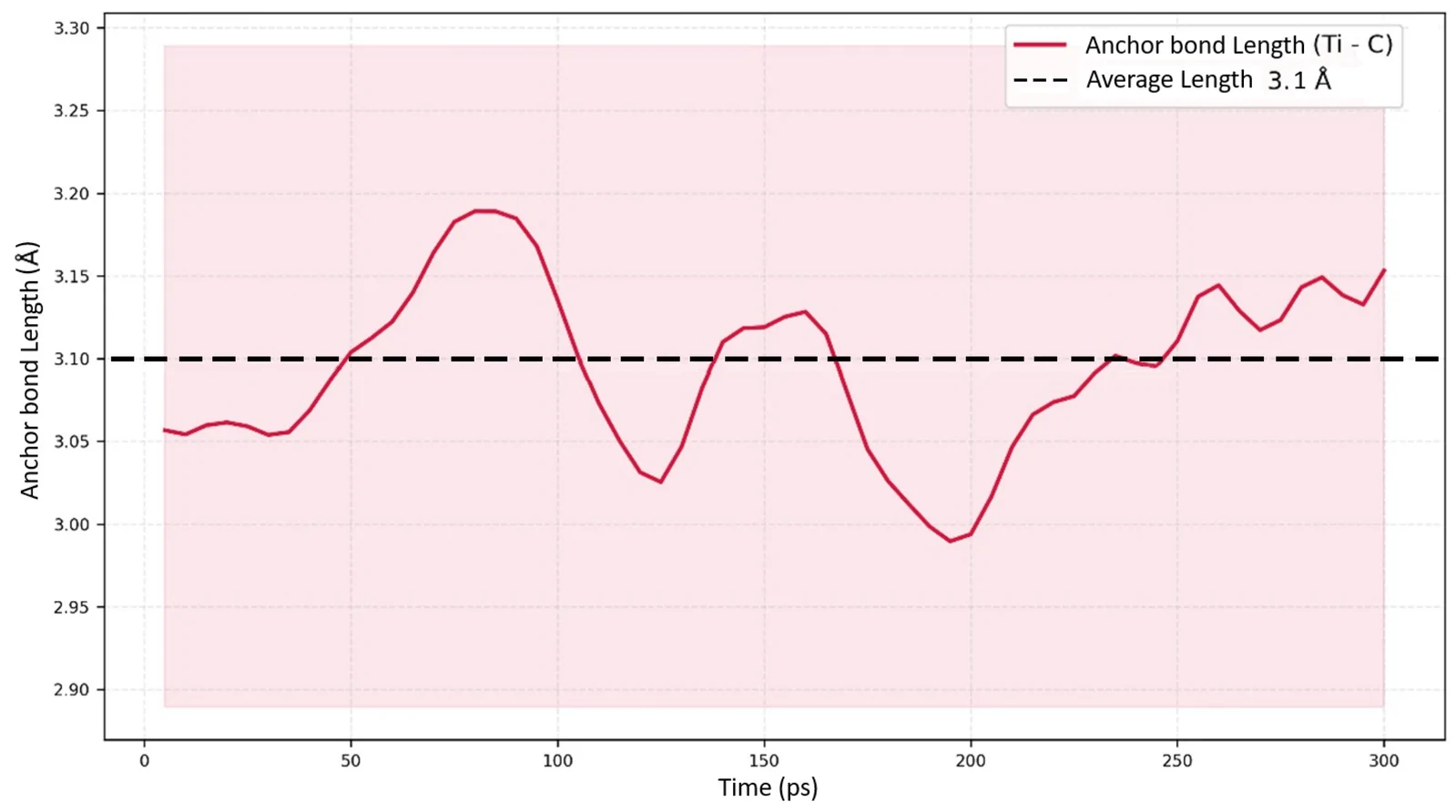
Structural stability of the GQD-TiO_2_ chemisorption bridge under thermal fluctuations.

Tracking the distance of the anchoring zone as a function of simulation time during the 300 K MD run demonstrated the complete structural integrity of the GQD-TiO_2_ composite. The length of the chemisorption bond oscillated harmonically around a mean value of approximately 3.1 Å, exhibiting absolutely no tendency toward bond cleavage (Figure 13). The preservation of stable anchoring under continuous thermal excitation proves that the energetic barrier to dissociation exceeds the available kinetic energy of the system at room temperature.

Consistent with established literature standards for semiconductor interface validation, the absence of dye desorption under lattice vibrations confirms that the adsorption zone resides in a deep energetic minimum. These results provide theoretical evidence for thermodynamic and operational robustness of the composite and therefore, the DSSC itself [54,67]. While these computational molecular dynamics simulations provide evidence for the thermodynamic resilience of the anchoring bonds against thermal desorption, evaluating the macroscopic long-term stability of the assembled device under continuous illumination remains a critical milestone. Future experimental investigations will focus on extended aging protocols paired with post-cycling destructive structural characterizations. These forthcoming studies aim to definitively confirm the resilience of the GQD/TiO_2_ interface against potential photo-degradation and continuous electrolyte exposure in fully scaled operational environments.

## 4. Conclusions

This study successfully demonstrated that microwave-assisted hydrothermal synthesis utilizing biodegradable precursors enables the efficient, eco-friendly production of highly luminescent graphene quantum dots. When implemented as sensitizers in Grätzel-type solar cells, these carbon nanostructures exhibited significant photoelectric activity across a broad spectral range (405–625 nm). The experimental evaluation revealed that the photovoltaic performance is strictly concentration-dependent, achieving a maximum open-circuit voltage ($V_{\mathrm{oc}}$) of 545.4 mV under UV irradiation at an optimal sensitizer concentration of 0.4 g/dm^3^ (1:5 dilution). Higher GQD concentrations (exceeding 1.0 g/dm^3^) were shown to induce excessive nanomaterial agglomeration, effectively blocking the mesoporous TiO_2_ network, hindering dye regeneration, and consequently degrading overall cell efficiency. Furthermore, the analysis of the dark voltage confirmed the exceptionally high chemical capacitance of the TiO_2_/GQD system, imparting electrochemical capacitor-like characteristics that enable the stabilization of electrical parameters under fluctuating illumination.

The atomistic computational models provided critical insights into these macroscopic observations. The simulations demonstrated that the stable chemisorption of GQDs onto the TiO_2_ surface via covalent Ti–O–C bridges is a prerequisite for effective interfacial electronic coupling and ultrafast charge injection. The integration of deprotonation and implicit solvent models further revealed that the aqueous electrolyte optimizes the energy band alignment, providing the necessary thermodynamic driving force for continuous dye regeneration. Moreover, molecular dynamics simulations at 300 K confirmed the thermodynamic resilience of the composite, proving that the anchoring bonds reside in a deep energetic minimum that ensures structural integrity without the risk of thermal desorption.

In conclusion, GQDs represent a safe and non-toxic alternative to heavy-metal-based sensitizers, aligning with current trends in sustainable materials engineering. The core contribution of this work lies in demonstrating that combining open-source experimental prototyping with advanced machine-learning models yields a comprehensive understanding of interfacial charge dynamics. Moving forward, future perspectives will focus on upgrading the custom experimental platform to enable full current–voltage (J-V) curve extraction and electrochemical impedance spectroscopy (EIS) under solar simulators. Additionally, long-term aging protocols under continuous illumination will be conducted to physically validate the macroscopic operational lifespan of the assembled devices, ultimately paving the way for the commercial scalability of these third-generation solar cells.

## 5. Patents

The method for synthesizing graphene quantum dots described in this article is the subject of a pending patent application.

## Figures and Tables

**Table 1:** Sample designations of the dye-sensitized solar cells relative to the dilution ratio of the graphene quantum dot stock solution and the corresponding mass concentrations.

No.	Sensitizer	Designation	Concentration [g/dm^3^]
1	GQDs–stock solution	1:1	2.0
2	GQDs–twofold dilution of stock solution	1:2	1.0
3	GQDs–fivefold dilution of stock solution	1:5	0.4
4	GQDs–tenfold dilution of stock solution	1:10	0.2
5	No sensitizer	pure TiO_2_	0.0

## Data Availability

The original contributions presented in this study are included in the article. Further inquiries can be directed to the corresponding author.
